# A Fast Storage Method for Drone-Borne Passive Microwave Radiation Measurement

**DOI:** 10.3390/s21206767

**Published:** 2021-10-12

**Authors:** Xiangkun Wan, Xiaofeng Li, Tao Jiang, Xingming Zheng, Xiaojie Li, Lei Li

**Affiliations:** 1 Northeast Institute of Geography and Agroecology, Chinese Academy of Sciences, No. 4888 Shengbei Street, Gaoxinbei District, Changchun 130102, China; wanxiangkun@iga.ac.cn (X.W.); zhengxingming@iga.ac.cn (X.Z.); lixiaojie@iga.ac.cn (X.L.); lilei@iga.ac.cn (L.L.); 2 College of Resources and Environment, University of Chinese Academy of Sciences, Beijing 100000, China; 3 Jingyuetan Remote Sensing Experimental Station, Chinese Academy of Sciences, No. 4888 Shengbei Street, Gaoxinbei District, Changchun 130102, China

**Keywords:** drone-borne microwave radiometer, SD card storage, STM32 MCU, time-sharing processing, dual polling

## Abstract

A drone-borne microwave radiometer requires a high sampling frequency and a continuous acquisition capability to detect and mitigate radio frequency interference (RFI), but existing methods cannot store such a large amount of data. In this paper, the dual polling write method (DPSM) for secure digital cards triggered by a timer under a multitask framework based on STM32 MCU is proposed to meet the requirements of continuous data storage. The card programming step was changed from a query waiting structure to a polling query flag bit structure, and time-sharing processing and parallel processing were used to simulate multithreading. The experimental results were as follows: (1) the time consumption of the whole storage procedure was reduced from 4000 microseconds to 200–400 microseconds; (2) the time consumption of the card programming step was reduced from 3000 microseconds in the first block and 1000 microseconds in the second and subsequent blocks to 17–174 microseconds and 18–71 microseconds, respectively, compared with the existing method; (3) the delay in the whole sampling cycle was reduced from 3942 microseconds to 0 microseconds. The results of this paper can meet the data storage requirements of a drone-borne microwave radiometer and be applied to the high-speed storage of other devices.

## 1. Introduction

A microwave radiometer is a high-sensitivity broadband noise receiver working in the microwave band, which can extract the variation in weak microwave radiation signals from strong background noise, and it mainly deals with the Gaussian white noise radiated by ground objects [1]. A spaceborne radiometer cannot be used for medium and small-scale applications due to its coarse resolution (≈25 km) [2,3]. The disadvantages of a ground-based radiometer [4,5] include its small observation range, poor mobility, and terrain limitation, while an airborne radiometer has the advantages of high spatial resolution and relatively large observation range, which fill the scale gap between space-based and ground-based radiation observations. In addition, the advantages of a drone-borne microwave radiometer include the convenient operation, strong real-time performance, and low cost [6,7], which can be used for brightness temperature observation at the block scale and can provide data support for soil moisture monitoring, snow observation model inversion, and satellite ground verification [8,9,10,11].

When a ground-based radiometer is used, electromagnetic signals with similar frequencies, usually from radar, cell phone base stations, automotive electronics, and other wireless devices can interfere with the signals received by the receiver [12,13]. In addition to these interferences, a drone-borne microwave radiometer can also be affected by electromagnetic interference coupled with a high-speed rotor coil and the internal vibration caused by an uneven rotor speed during flight. For rapidly changing ground object targets, in order to ensure data quality and to detect and mitigate interference [1,14,15,16,17], reducing the integration time and increasing the sampling frequency are necessary, and they lead to a substantial increase in the amount of data. When considering power consumption and stability, having sufficient on-board storage is the best way to ensure complete recording of data.

At present, the microcontrollers launched by various chip manufacturers are integrated with rich peripheral resources, such as SPI, I2C, SDIO, CAN, Ethernet, etc. The early mature peripherals (USART and SPI) are relatively simple to control and use, while the SDIO and Ethernet with relatively complex control protocols require multiple control steps to complete one operation, and the control consists of mostly ‘command–response’ operations. Even with DMA, many valuable CPU cycles are required to query the status of each step. The radiometer used in this study was designed based on an STM32 micro control unit (MCU). There are two communication protocol modes in a secure digital (SD) card based on a STM32 processor: namely, the SPI mode and the SD mode [18]. Writing to the drive is simple in the SPI mode, and the host uses the SPI bus to access the card. Limited by its hardware structure, the transmission rate is low, so the SPI mode is suitable for tasks with short development cycles and small amounts of storage and transmission data [19,20,21]. In contrast, the SD mode has complex drivers and numerous internal functions, but its transmission rate is fast and its performance is stable, which makes it suitable for projects with a large amount of data storage [22,23]. Therefore, the SD mode is selected for this study for its higher transmission rate.

The MCU is connected to the SD card through secure digital input and output (SDIO) and is generally transmitted in the form of a data block. The main operations include an initialization parameter setting, a power-on status query, data block read and write operations, etc. The SD card initialization parameter setting and power-on status query process can be referenced in the official manual [24]. Data block reading and writing includes single-block or multi-block reading and writing. The block writing procedure consists of five steps: setting the block size, obtaining the card status, setting the write address, selecting and enabling the device mode, and waiting for the card programming to finish. The device mode can select the polling mode, the interrupt mode, and the direct memory access (DMA) mode. 

The polling mode occupies the most MCU time compared with the other two modes, which is not suitable for the multitasking framework of this study; the interrupt mode also takes up more time and has a medium speed; while the DMA mode copies data from one address space to another. When the CPU initializes the transfer action, the transfer action itself is performed and completed by the DMA controller. Using DMA does not delay the processor’s work and can be rescheduled to accommodate other work [25]. Therefore, SDIO + DMA is suitable for high-speed SD card storage in this study.

**Figure 1:**
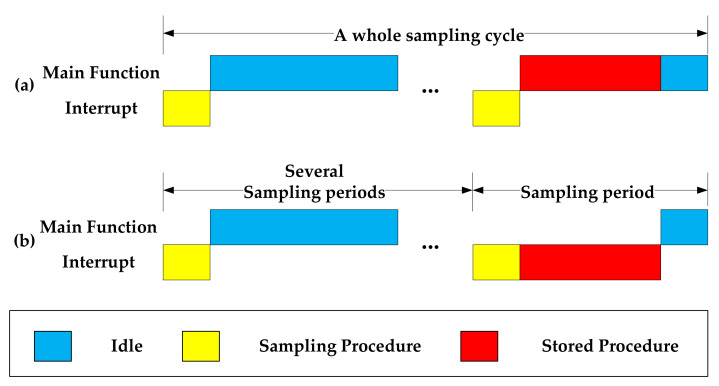
The time sequence diagram of low-speed sampling tasks for (**a**) storage in the main function loop and (**b**) storage in the interrupt.

From the perspective of hardware, a STM32 single core MCU can only run within a single thread at a time. Storage operations usually need to be carried out after acquisition and processing processes are stopped. For tasks in which the storage time consumed is less than the idle time, we define them as low-speed sampling tasks:

T_Stored_ < T_IDLE_ = T_Per_ − T_Sam_, (1)

where T_Stored_ and T_Sam_ are the time consumptions of the stored procedure and sampling procedure, respectively; T_Per_ is the sampling period, and T_IDLE_ is the idle time of a single sampling period. A continuous time sequence in the main function infinite loop or the interrupt is usually allocated to complete the storage operation between two acquisitions. Since the storage time is less than the idle time of a single sampling period, the whole sampling cycle will not be delayed. This method is simple, and the development cycle is short. Therefore, many devices adopt this method [18,26]. After a certain number of acquisitions, the data are encoded and stored. The time sequence is shown in Figure 1.

However, this method is not suitable for a drone-borne radiometer, which requires continuous and rapid data acquisition for time domain pulse detection in order to detect and mitigate RFI because the idle time of a single sampling cycle is far less than the storage time. If the acquisition is temporarily stopped for storage by turning off the timer that triggers acquisition or storage in the interrupt, taking higher priority, a delay will occur in the whole sampling period

T_Delay_ = T_Stored_ − T_IDLE_. (2)

**Figure 2:**
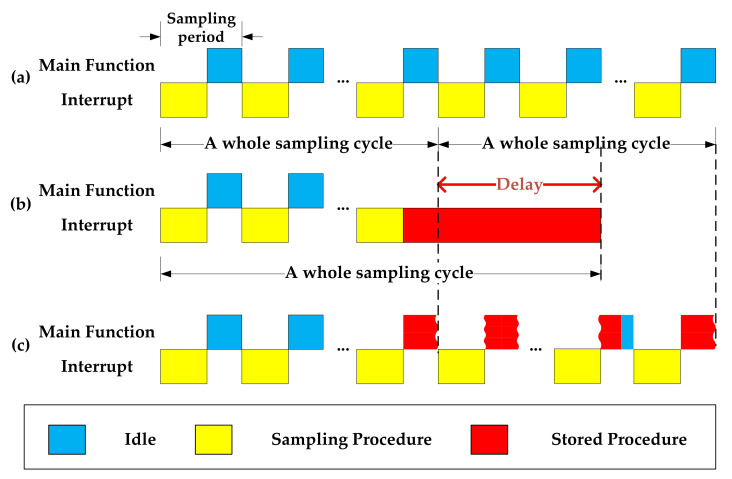
The time sequence diagram for (**a**) no storage operation is performed, (**b**) storage in the interrupt, and (**c**) storage in the main function loop.

As shown in Figure 2b, the task requirements of continuous sampling are not met. In contrast, if the acquisition process is not suspended as shown in Figure 2c, namely, carried out in the main function loop, the program will enter the timer interrupt frequently due to the high AD sampling frequency connected to the SPI port, which may cause the storage procedure to be interrupted frequently and can cause program exception. If the whole sampling cycle time is less than the time required for storage, the data from the previous cycle will not have been stored, and the data collected in the following cycle will need to be stored. Then, the data cache is blocked, which causes a program crash and does not meet the design requirements.

This paper analyzes the impact of existing storage methods, the main function storage method (MFSM), and the interrupt-triggered storage method (ITSM), on the radiometer acquisition process and the underlying functions of each step in the write procedure, despite other kinds of processing chips being used, such as STM32f4 series, which was faster, or FPGA, with parallel and pipelined technology [27,28]; the same problem is encountered when MFSM and ITSM are used for storage after data acquisition with high sampling frequency, but their threshold is higher. Therefore, a new time-sharing processing fast storage method for SD cards, a dual polling storage method (DPSM) triggered by timer interrupt under a multitask framework based on STM32 MCU, is proposed to realize the fast SD storage of a drone-borne microwave radiometer under the requirement of continuous acquisition. The research also has a certain role in promoting the multiperipheral, multitask, and high-speed cooperative work of various microcontrollers.

## 2. Materials and Methods and Experiments

### 2.1. Dual Polling Storage Method (DPSM) Triggered by Timer Interrupt

In the case of fast acquisition, using MFSM would cause frequent timer interruptions and program exceptions. While using ITSM could avoid this problem and improve the stability of the program, it will cause delays and cannot meet the task requirements of continuous acquisition. To solve the problems mentioned above, this study designed and built a hardware test environment to monitor each step of the single-block writing procedure. Based on the results of statistical analysis, a new and improved method, DPSM, is proposed. The framework was redesigned, and the underlying algorithm function of the single-block writing procedure was scattered and reorganized to provide a stable, reliable, and high-speed SD card storage scheme.

The study used time-sharing processing technology to divide the run time of the processor into very short time slices, reasonably arranging the timing of the sampling task, the data processing task, the data coding task, and the data storage task; made full use of the resources; and improved the utilization rate of resources. At the same time, the idea of the web solution polling structure was applied to the program. With the help of the DMA device mode, the STM32 MCU with a single core and a single thread was simulated as multithreading.

#### 2.1.1. The Framework and Time Sequence of DPSM

**Figure 3:**
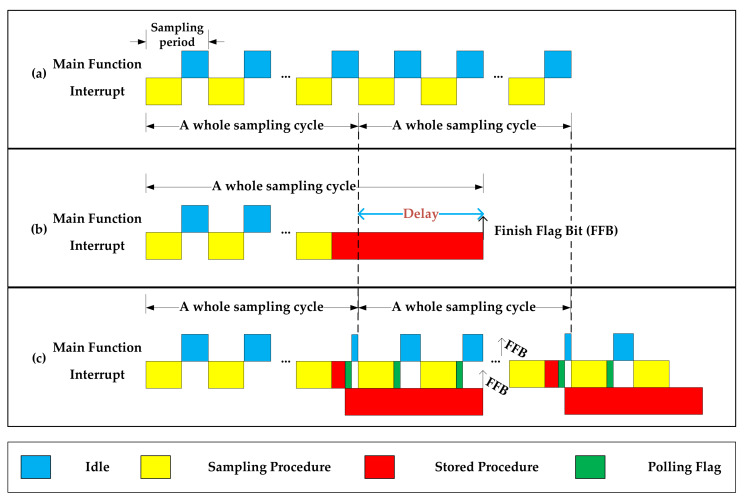
The time sequence diagram (**a**) when no storage operation is performed, (**b**) for ITSM, and (**c**) for DPSM.

Benefitting from the DMA device mode adopted by the SD card storage, the card programming step was directly controlled by the SD card without the participation of the main function. The SD card can be regarded as another core that provides the possibility of reconstruction of the underlying framework of the block write operation. Compared with ITSM shown in Figure 3b, the main improvement was the reconstruction of the underlying logic of the writing procedure; that is, the card programming step was changed from the query waiting order structure to the polling query flag bit structure, and time-sharing processing and parallel processing were used to simulate a single thread as multithreading, as shown in Figure 3c. As seen when comparing Figure 3b,c, the delay caused by storage operation could be eliminated using this method.

Polling was originally a web application scheme. The client sent requests to the server at a certain time interval to solve the problem of data synchronization. In the first polling, the main function, as a server, sent a request to query flag bits after a time interval of the whole sampling cycle to judge whether the writing process is completed. In the second polling, the timer interrupt was used as a server; it sent a request to query flag bits after a time interval of a sampling period to determine the progress of the stored procedure and check the card status to determine whether the card programming step was complete.

#### 2.1.2. Applicable Conditions and Theoretical Time Consumption

For the convenience of description, the first four steps (setting the block size, obtaining the card status, setting the write address, and selecting and enabling the device mode) in each block write procedure were defined as Procedure 1, and the card programming step, which takes a longer time, was defined as Procedure 2; then, we have the time consumed by the stored procedure:

T_Stored_ = T_Pro1_ + T_Pro2_, (3)

and

T_Stored_ < T_Cycle_, (4)

where T_Pro1_, T_Pro2_, and T_Cycle_ are the time consumption of Procedure 1, Procedure 2, and the whole sampling cycle, respectively.

Due to the polling mode adopted by Procedure 2, it can be directly entered into Procedure 2 for query after Procedure 1 is completed, rather than jumping out of the interrupt and waiting for the next sampling period for judgment, so that each write procedure can reduce the number of interrupt judgments by at least one sampling period and can reduce the burden of interrupt control. A query is performed to check if Procedure 2 finished after each sampling instead of waiting, and its time consumption is T_Query_; then, we have the new time consumption

(5) $${T’}_{\mathrm{Stored}}=T_{\mathrm{Pro}1}+{T’}_{\mathrm{Pro}2}=T_{\mathrm{Pro}1}+{Ʃ}_{i=0}^{N}\;T_{\mathrm{Query}\;i,}$$

where N is the number of queries,

N = round down {[T_Pro2_ − (T_Per_ − T_Sam_)] × (T_Per_)^−1^} + 3. (6)

#### 2.1.3. The Logic Flow Chart and Algorithm of DPSM

An idle time sequence after collection is specially allocated for transcoding in the main function to avoid small delay caused by too many operations in the same time sequence. Considering that the time consumption of the storage procedure for the first block differed from the others, writing more than two blocks was the same in principle, so two data blocks written at a time was chosen as an example.

**Figure 4:**
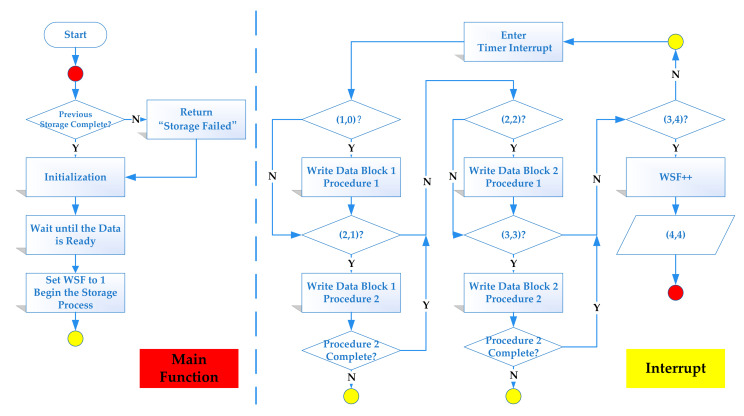
The storage logic flow chart of DPSM of storing two data blocks.

Figure 4 is the storage logic flow chart of DPSM. The framework itself formed a closed loop and had good robustness. It had the ability to correct abnormal events and handle emergent situations. We define two flag bits: the write status flag (WSF) and the write finish flag (WFF). For the convenience of description, the framework flow chart in this paper used the following convention: I and J in the structure (I, J) represent the values of the WSF and WFF, respectively. For example, the state (4,4) indicates that the write status flag bit was 4 and that the write finish flag bit was 4, which was also a flag that the block write process was completed. The logic of the flow chart was as follows.

(a) The main program polled the write status flag bit to determine whether the previous stored procedure was completed (WSF = 4?). If the procedure was not completed, the procedure had timed out; otherwise, it would be initialized, and the flag bit would be cleared.
(b) Wait until the data acquisition was ready, complete the coding, and set the WSF.
(c) When the main function infinite loop was running, the program would trigger the timer for interrupt when the counter of the timer accumulated a certain value, and the update flag would then be set. The main function would poll whether the WSF is set each time the interrupt was entered. If set, the state should be (1,0), and the next operation, step d, would be performed; otherwise, wait for the next timer interrupt. The timer interrupt threshold should be set to the frequency of AD sampling.
(d) The WSF increased automatically and performed Block 1 Procedure 1. After the process was completed, the WFF increased automatically and initiated the next operation. At that time, the state should be (2,1).
(e) When the state was (2,1), Block 1 Procedure 2 should be performed. The WFF was polled to determine whether card programming had finished. If finished, the WFF increased; the state should be (2,2); and the next operation, step f, should be performed. Otherwise, exit the interrupt and wait for the next interrupt.
(f) Repeat the above steps for data Block 2. The state should be (3,4) after the stored procedure finishes.
(g) Poll the flag bit until all of the above steps are completed and the WSF increased automatically. At this time, the status is (4,4), and all of the stored procedures are completed. Then, returned to step a.

### 2.2. Comparative Experiments

**Figure 5:**
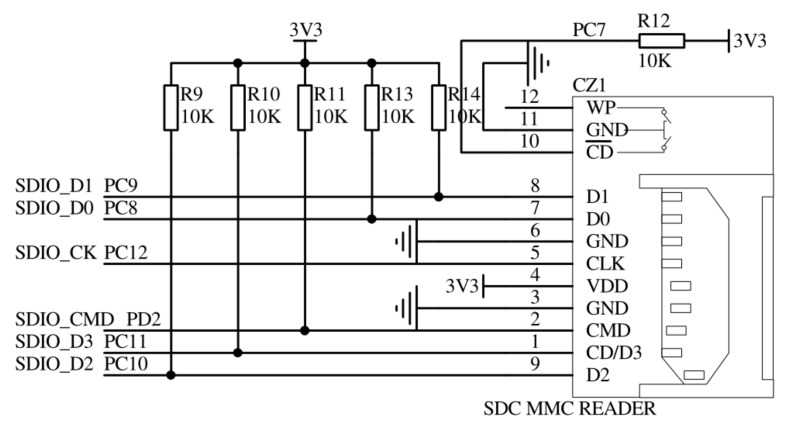
SDIO interface schematics.

In this study, the ARM Cortex-M3 core 32-bit high-performance microcontroller STM32F103ZET6 chip was selected as the micro control unit. The maximum internal working clock frequency of the chip could reach 72 mhz. SPI, USART, SDIO, DMA, and other peripherals were integrated in the chip, which could be called directly to facilitate development. The hardware running environment of the experiment was built by connecting the corresponding devices through the development board. The connection circuit of the SDIO interface is shown in Figure 5.

The developmental environment of the STM32 micro control unit was the RealView microcontroller development kit (MDK), and the version was Keil μVision5, which was an embedded software development tool for ARM processors launched by the ARM company based on μVision interface, which provided a perfect C/C++ development environment. Additionally, the J-LINK V9 downloader was used to download the code to flash memory.

In this study, two groups of experiments were carried out. The first experiment in Section 2.2.1 was to monitor the time consumption of all of the procedures of each data block, and there was no acquisition process. The purpose was to analyze and locate the reasons for the long duration of the storage operation and to count the probability and distribution of abnormal time consumption of each process. The second experiment in Section 2.2.2 aimed to verify the performance of the three storage methods under the multi-acquisition task. The experiment was carried out on the PCB with the same configuration as the microwave radiometer. Since the front end of a radiometer had not been debugged, the SPI pin connected to ADC (ad7866 used in this paper) was temporarily NC (not connected), without signal input. In fact, the acquisition was still carried out, the working state and other process were the same as that of microwave radiometer except for the front end that really worked and that would not be affected by the key link of this study—later data acquisition and processing. Therefore, the experiment in Section 2.2.2 collects real data rather than simulated data.

#### 2.2.1. Time Consumption of Single-Block Writing Steps

The block writing procedure consisted of five steps: setting the block size, obtaining the card status, setting the write address, selecting and enabling the device mode, and waiting for card programming to finish. By monitoring the corresponding time consumption of these five steps of a single-block write procedure, a set of consecutive single-block write experiments was designed to explore the impacts of data length and block writing order on the write time.

The block size of each data block was 512 bytes. The impact of the data length on the write time was explored by comparing the write time of each single data block. The impact of the block writing order was explored by comparing the time consumption of each step of each block, and whether the impact of the writing order on the time consumption was caused by the physical layer writing mechanism could be determined through the time consumption comparison among the first block and the second and subsequent blocks.

#### 2.2.2. Performance Verification of Different Write Methods

By monitoring the overall and the various steps of the storage procedure time consumption, the delay in the storage procedure to the whole sampling cycle, as well as the time consumption of the sampling procedure, a set of comparative experiments was designed to explore the impact of different sampling intervals on MFSM, ITSM, and DPSM, and their applicable environment under the multi-acquisition task framework. 

The data were stored once every 1000 instances of sampling into two data blocks after integration and coding. Three general-purposed timers were used in the experiments, TIM2 was used to trigger AD sampling at a short time interval, TIM3 was used to trigger parameter sampling, which did not require frequent sampling, such as temperature, at a relatively long time interval, and TIM4 was used to monitor the above parameters, which would be sent to the host computer through a serial port for statistical analysis. The sampling period or the update cycle of the timer interrupt could be controlled by changing the value of the prescaler register (PSC) and auto-reload register (ARR). The following experiments were carried out.

(a) The overall time consumption and success rate of the three methods under different sampling interval conditions were monitored to find their applicable environments.
(b) The time consumption of each step of DPSM under different sampling interval conditions was monitored in order to verify the proposed method.
(c) The delay caused by the storage procedure to the whole sampling cycle can be calculated by monitoring the time consumption of the corresponding position of the sampling cycle when storing or not storing.

## 3. Results and Discussions

### 3.1. Time Consumption Results of Single-Block Writing Steps

**Figure 6:**
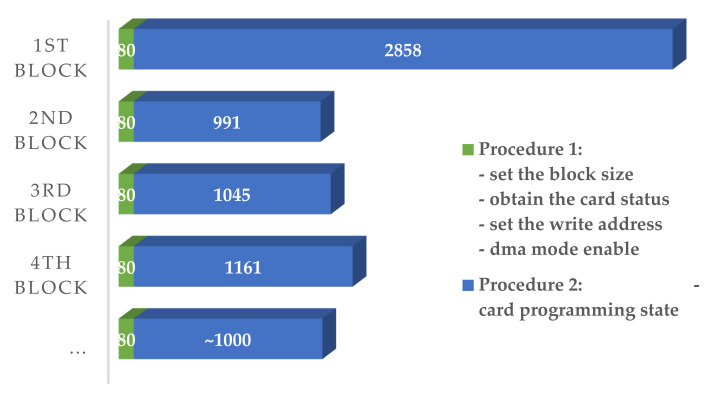
Histogram of the typical value distribution of the time consumption of each write procedure (in microseconds).

The typical time consumption of each step of the consecutive single-block writing experiment is shown in Figure 6. Procedure 1 was composed of four steps: setting the block size, obtaining the card status, setting the write address, and DMA mode enabling, consuming a total of 80 microseconds. The first three steps consumed no more than 10 microseconds. The DMA transmission step, which sent data and parameters from the host to the SD card cache, consumed approximately 52 microseconds. Compared with the sampling period, these steps had a high probability of not affecting the process. Additionally, the last step of the card programming process (Procedure 2) consumed approximately 3000 microseconds for the first block and 1000 microseconds for the second and subsequent blocks, which was significantly less than that of the former. From the results of the experiment, we found that (1) the data length affected the total write time and the average write time of each block, in which the longer the data, the shorter the average time; (2) due to the SD card’s physical layer driver or other reasons, the write time mainly depended on whether the write order is the first block; and (3) the write time was hardly affected by the content.

The time consumption of each step may exceed the typical value (±1 microsecond) during a mass write stress test, and the statistical results are shown in Table 1. The probability of a timeout occurrence of the first four steps was no more than 0.04‰, and the timeout was less than 100 microseconds, which had little impact on the write procedure, and most task requirements can be met by certain restrictions. The time consumption of the card programming showed strong volatility and a proportion far beyond the typical value range (more than 10 milliseconds), which could reach 1.75‰.

The card programming step accounted for more than 92% of the whole stored procedure (Figure 6), which was the key object to be studied. At the same time, if a larger buffer was set up, the average time consumption of centralized writing for each block would be reduced. However, due to the limitation of high sampling frequencies of multitasking frameworks, no large amount of continuous time sequence was allocated to SD card storage procedures. Therefore, the structure of the card programming was reconstructed to reduce the storage time consumption, and DPSM was proposed.

### 3.2. Performance Result of Different Write Methods

A large number of block writing experiments have been conducted. The overall and various steps of the stored procedure time consumption and the delay in the storage procedure for the whole sampling cycle were monitored. The performance and applicable environment of the three storage methods, MFSM, ITSM, and DPSM, under the multi-acquisition task framework were verified.

(a) Total Time Consumption and Success Rate

The test results of the total time consumption T_Stored_ and success rate of the three storage methods under different sampling intervals are shown in Table 2. The results show the following: (1) MFSM was only suitable for tasks with a few acquisition tasks of which the sampling period was greater than the sum of the storage time and sampling time. In this experiment, each sampling procedure took about 82 microseconds, and the storage time consumption was about 4000 microseconds. In order to ensure the success rate of storage and the robustness of the program, the sampling period was recommended to be greater than 10 milliseconds. This method would not cause delay to the whole sampling cycle. Continuous and fast sampling would frequently interrupt the main program process, cause data blocking, and finally lead to program exception. (2) ITSM was applicable to tasks with a sampling period no less than 170 microseconds when performing dual acquisition tasks. A delay of about 4000 microseconds would be seen in the whole sampling cycle, and this delay was hardly affected by the sampling period. (3) The improved writing method DPSM in this study was also suitable for tasks with a sampling period of no less than 170 microseconds when performing dual acquisition tasks. The storage procedure took a total of 200–400 microseconds, which was negatively correlated with the sampling period. Compared with ITSM, the storage operations were scattered within the idle time of each sampling period, and the time consumption of each operation was less than that idle time; thus, the whole sampling cycle would not be delayed. No matter how many acquisition tasks were added, DPSM can complete the storage task well as long as the acquisition time did not exceed the idle time of a single sampling period. In addition, the storage time was limited to within 70 sampling periods in this study, for we preferred to allocate the rest of the idle time (930 of 1000 sampling periods) to other operations, resulting in some ‘unsuccessful’ storage, which would cause some data loss. Theoretically, the limit could be up to the whole sampling cycle time, which could basically avoid this phenomenon. If the event did occur, the probability of occurrence was less than 0.2%, and a very small amount of data would be lost. However, the results of applications such as radio frequency interference detection of drone-borne microwave radiometer data and surface parameter inversion would not be affected.

(b) Time Consumption of Two Procedures of DPSM

The test results of the time consumption of two procedures of DPSM under different sampling intervals are shown in Table 3. For different sampling periods, the time consumption of Procedure 1 for each write command remained unchanged, and the time consumption of Procedure 2 was negatively correlated with the sampling period, which was the main reason for the difference in total time consumption. The threshold of the sampling period needed to be greater than the sum of the time-consuming of the sampling procedure and Procedure 1 to ensure that the whole sampling cycle would not be delayed and that the acquisitions were uniform and continuous.

Compared with the MFSM and ITSM, the time consumption of Procedure 2 was reduced from about 3000 microseconds (seen in Figure 6) to 17–174 microseconds, which was 0.6–6% for the first block, and from about 1000 microseconds (seen in Figure 6) to 18–71 microseconds, which was 2–7% for the second and subsequent blocks. Additionally, these processes were carried out during the idle time of the sampling period, which would not cause delay to the whole sampling cycle. Note that the time consumption of Procedure 2 here refers to the integration time of several times querying whether Procedure 2 was completed, rather than the actual time consumption of Procedure 2. After the completion of Procedure 1, each time the timer interrupt was triggered, a query of whether Procedure 2 was completed would initiate, which takes about 8 to 9 microseconds. As for the total DMA time that was almost constant, the longer sampling period meant a longer interval between two queries, that is, the longer the DMA run time, which resulted in fewer queries and less time consumed by Procedure 2.

In addition, the time consumed for the interrupt query can be further limited in the program. For example, the query was performed once every several interruptions, which can reduce the number of queries N to reduce the integration time, and the time saved can be used to carry out more other alternative operations. If an inappropriate value was selected, the delay might become longer, which may occur in some extreme settings. For most cases, we recommend setting this value to 10, which can reduce the Procedure 2 time consumption the most: to within 20 microseconds, that is, up to 11% of the original time.

(c) The Delay to the Whole Sampling Cycle Caused by the Stored Procedure

**Figure 7:**
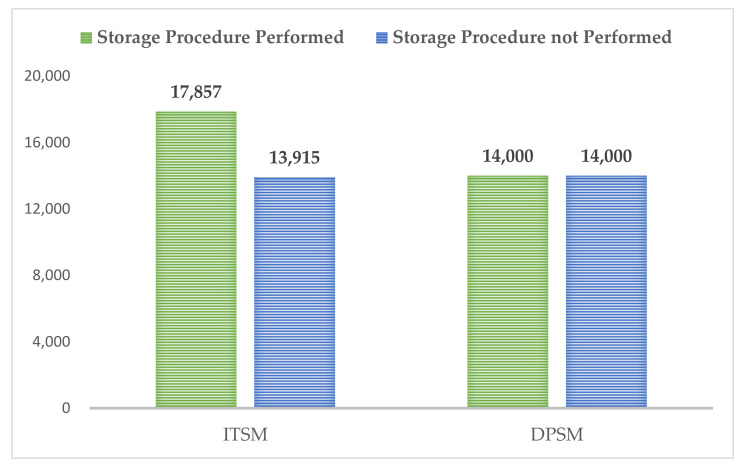
Comparison of the average time consumption between whether to perform storage procedure for ITSM or for DPSM (in milliseconds).

The delay to the whole sampling cycle caused by the stored procedure was calculated by monitoring the time consumption of the corresponding locations of the sampling cycle when ITSM and DPSM were applied. The results are shown in Figure 7. The time consumption of the corresponding position for ITSM increased from 13,915 microseconds when no storage operation was performed to 17,857 microseconds when a storage operation was performed, resulting in a delay of 3942 microseconds. As for DPSM, the time consumption of the corresponding position remained unchanged at 14,000 microseconds regardless of whether a storage operation was performed, without causing any delay, which was in line with our expected assumption.

In addition, some steps in Procedure 1 could also be changed from the query waiting order structure to the polling query flag bit structure, and the time consumption can be further reduced, but the improvement was not very large.

## 4. Conclusions

For the continuous acquisition storage problem based on STM32 MCU, this paper studies the advantages and disadvantages as well as the applicable conditions of the existing storage method, and it proposes a new fast storage method: the dual polling write method (DPSM) for SD card triggered by timer under a multitask framework. The time-sharing processing technology and the polling structure are effectively used to realize stable, reliable, and high-speed storage, which solves the problem of data storage during continuous acquisition of a drone-borne passive microwave radiation measurement. Experiments were designed, and the stability and feasibility of the framework were verified on the hardware test environment. Compared with the existing methods, the storage procedure does not delay the whole sampling cycle, the overall time consumption is reduced by 90–95%, and the time consumption of the card programming step is reduced by 93–99%, which greatly saves the time sequence occupied by storage. 

The proposed method should be applied to a drone-borne microwave radiometer to ensure that the radiometer carries out equal-interval high-frequency sampling in time as much as possible, which ensures the spatial continuity of ground data to a great extent, and it is very helpful for subsequent data processing and image mosaic. The dual polling storage method itself forms a closed loop and has the ability to correct for abnormal events and to handle emergent situations, with good robustness. The storage method has good portability and can be applied to improve the efficiency of data storage under a multitasking framework besides for a drone-borne radiometer. For better chips, this method could further improve storage capacities.

## Figures and Tables

**Table 1:** Proportion of typical value and timeout value of each step.

Steps	Set the Block Size	Obtain the Card Status	Set the Write Address	DMA Mode Enable	Card Programming
Proportion of typical value (‰)	999.989	999.989	999.993	999.962	998.243
Proportion of timeout value (‰)	0.011	0.011	0.007	0.038	1.757

**Table 2:** Total time consumption and success rate of three storage methods under different sampling intervals in a multi-acquisition task framework.

TIM2 Sampling Period	TIM3 Sampling Period	Number of Experiment Repeats	MFSM		ITSM		DPSM	
			Success Rate (%)	T_Stored_ (us)	Success Rate (%)	T_Stored_ (us)	Success Rate (%)	T’_Stored_ (us)
>10 ms	10 s	1000	100	3942.1	100	3946.7	100	200.7
1 ms	1 s	2000	0	/	100	3917.8	100	220
200 us	200 ms	5000	0	/	99.82	3904.3	99.8	358.9
180 us	180 ms	5000	0	/	99.82	3942.5	100	379.1
170 us	170 ms	5000	0	/	99.76	3967	99.96	390.1
160 us	160 ms	5000	0	/	0	/	0	/

**Table 3:** The time consumption of each step of DPSM under different sampling intervals.

TIM2 Sampling Period	TIM3 Sampling Period	Total Duration (us)	Block 1 Procedure 1 (us)	Block 1 Procedure 2 (us)	Number of Queries	Block 2 Procedure 1 (us)	Block 2 Procedure 2 (us)	Number of Queries
10 ms	10 s	200.7	82	17	2	82	18	2
1 ms	1 s	220	82	44	5	82	26	3
200 us	200 ms	358.9	82	143	17	82	53	6
180 us	180 ms	397.1	82	153	18	82	62	7
170 us	170 ms	390.1	82	160	19	82	71	8

## Data Availability

Not applicable.

## Abbreviations

The following abbreviations are used in this manuscript:

RFI	Radio Frequency Interference
DPSM	Dual Polling Write Method
MCU	Micro Control Unit
SD	Secure Digital
SDIO	Secure Digital Input and Output
DMA	Direct Memory Access
MFSM	Main Function Write Method
ITSM	Interrupt-Triggered Storage Method
WSF	Write Status Flag
WFF	Write Finish Flag
MDK	Microcontroller Development Kit
PSC	Prescaler Register
ARR	Auto-Reload Register
